# Ancestral Wheat Types Release Fewer Celiac Disease Related T Cell Epitopes than Common Wheat upon Ex Vivo Human Gastrointestinal Digestion

**DOI:** 10.3390/foods9091173

**Published:** 2020-08-25

**Authors:** Tora Asledottir, Rashida Rehman, Gianfranco Mamone, Gianluca Picariello, Tove Gulbrandsen Devold, Gerd Elisabeth Vegarud, Arne Røseth, Tor Erling Lea, Trond S. Halstensen, Pasquale Ferranti, Anne Kjersti Uhlen

**Affiliations:** 1 Faculty of Chemistry, Biotechnology and Food Science, Norwegian University of Life Sciences, 1433 Ås, Norway; rashida.rehman@nmbu.no (R.R.); tovede@nmbu.no (T.G.D.); gerd.vegarud@nmbu.no (G.E.V.); tor.lea@nmbu.no (T.E.L.); 2 Institute of Food Science, National Research Council, 83100 Avellino, Italy; mamone@isa.cnr.it (G.M.); picariello@isa.cnr.it (G.P.); ferranti@unina.it (P.F.); 3 Department of Internal Medicine, Lovisenberg Diaconal Hospital, 0456 Oslo, Norway; arneroseth@gmail.com (A.R.); t.s.halstensen@odont.uio.no (T.S.H.); 4 Department of Oral Biology, Faculty of Dentistry, University of Oslo, 0372 Oslo, Norway; 5 Department of Agriculture, University of Naples Federico II, 80055 Portici, Italy; 6 Faculty of Biosciences, Norwegian University of Life Sciences, 1433 Ås, Norway; anne.uhlen@nmbu.no

**Keywords:** Celiac disease, wheat, ex vivo digestion, T cell epitope, immunogenic peptide

## Abstract

Celiac disease (CeD) is an autoimmune enteropathy triggered by immunogenic gluten peptides released during the gastrointestinal digestion of wheat. Our aim was to identify T cell epitope-containing peptides after ex vivo digestion of ancestral (einkorn, spelt and emmer) and common (hexaploid) wheat (Fram, Bastian, Børsum and Mirakel) using human gastrointestinal juices. Wheat porridge was digested using a static ex vivo model. Peptides released after 240 min of digestion were analyzed by liquid chromatography coupled to high-resolution mass spectrometry (HPLC-ESI MS/MS). Ex vivo digestion released fewer T cell epitope-containing peptides from the ancestral wheat varieties (einkorn (*n* = 38), spelt (*n* = 45) and emmer (*n* = 68)) compared to the common wheat varieties (Fram (*n* = 72), Børsum (*n* = 99), Bastian (*n* = 155) and Mirakel (*n* = 144)). Neither the immunodominant 33mer and 25mer α-gliadin peptides, nor the 26mer γ-gliadin peptide, were found in any of the digested wheat types. In conclusion, human digestive juice was able to digest the 33mer and 25mer α-gliadin, and the 26mer γ-gliadin derived peptides, while their fragments still contained naive T cell reactive epitopes. Although ancestral wheat released fewer immunogenic peptides after human digestion ex vivo, they are still highly toxic to celiac patients. More general use of these ancient wheat variants may, nevertheless, reduce CeD incidence.

## 1. Introduction

Wheat proteins can trigger hypersensitivity reactions such as allergy or intolerance. Celiac disease (CeD) is an autoimmune hypersensitivity reaction induced by wheat gliadins in genetically susceptible individuals. Population screening has revealed that the prevalence of CeD is 1–2% in Europe and the United States [1,2], although many patients remain undiagnosed [3]. In addition to wheat gluten (gliadin and glutenin), CeD patients react to structurally related gluten proteins in rye (secalins), barley (hordeins) and in extremely rare cases from oat (avenins) [4]. The gluten-induced intestinal inflammation leads to small intestinal crypt cell hyperplasia, villous atrophy, and as a consequence, nutrient malabsorption [5]. The dominating genetic susceptibility to CeD is to carry the human leukocyte antigen (HLA) DQ-2.5 or DQ8 alleles [6]. Globally, approximately 90% of CeD patients express the HLA-DQ 2.5 heterodimer (composed of HLA-DQA*0501 and HLA-DQB*0201), whereas the rest predominantly express HLA-DQ8, with only a few patients expressing one of the HLA-DQ-2.5 chains either in the HLA-DQ2.2 or -DQ7.5 alleles [7]. Although HLA is the most important and necessary genetic risk factor, more than 39 other loci are associated with CeD development [8].

The CeD-associated HLA-DQ molecules bind gliadin peptides that have been deamidated by the autoantigen tissue transglutaminase-2 (tTG2) with high affinity. However, the selection of gluten reactive T cell epitopes depends on at least three factors: (1) resistance to proteolytic digestion, (2) specificity for tTG2 deamidation and 3) HLA-DQ-2.5/8 binding properties.

The major protein groups in wheat are the storage proteins gliadin and glutenin, which make up 80% of the total grain protein in common wheat [9]. These proteins are rich in glutamine and proline, which makes gluten highly resistant to gastrointestinal proteolytic degradation, resulting in long peptides ranging from 15 to 50 residues [10]. An example is the well-known 33mer α-gliadin fragment (LQLQPFPQPQLPYPQPLPYPQPQLPYPQPQPF) that has been considered to be one of the main culprits in CeD [10,11] as it contains six overlapping T cell epitopes. However, several other long gluten peptides containing CeD-associated T cell epitopes have been identified [12,13,14].

Common bread wheat (*Triticum aestivum*) has evolved from hybridization between the tetraploid species *T. turgidum* (AABB) and the diploid species *Aegilops tauschii* (DD) [15]. The common hexaploid wheat (AABBDD) adapts well and grows more robustly, as well as having a favorable gluten composition for industrial quality compared to its ancestors [16]. However, the difference in gluten composition among diploid (AA), tetraploid (AABB) and hexaploid (AABBDD) wheat varieties may affect digestion-induced cleavage [17]. Substituting einkorn (*Triticum monococcum*) for common wheat may delay CeD onset in HLA-DQ2.5/-8 positive first-degree relatives because it contains fewer putative T cell epitopes [14] (partly because diploid and tetraploid wheat lacks the D genome, where the α-gliadin 33mer is located).

The aim of this study was to compare peptide profiles and identify gluten peptides produced ex vivo from seven different wheat types using human gastrointestinal juices. The digestion was performed with ancestral wheat (einkorn, spelt and emmer) and varieties of common wheat cultivated in Norway during 1900–1930 (Børsum, Fram) and 1990–2020 (Bastian, Mirakel). As the activity and specificity of digestive enzymes, including trypsin, differ among sources [18], the use of human digestive enzymes will be a more correct representation of the in vivo situation compared to enzymes of bovine or porcine origin, which have been applied in most in vitro studies published to date.

Digestion-released peptides were identified by high performance liquid chromatography/high-resolution electrospray ionization/tandem mass spectrometry (HPLC-ESI-MS/MS) analyses. The peptides were compared to the nine amino acid core region recognized by CeD-associated CD4+ T cells [19]. The 33mer and 25mer α-gliadins, and the 26mer γ-gliadin immune dominant peptide, were further monitored, as they consist of several overlapping T cell reactive epitopes.

## 2. Materials and Methods

### 2.1. Wheat Sample Collection

Wheat was collected in 2017 from an experimental field (Vollebekk Research Farm, Norwegian University of Life Sciences, Ås, Norway). All wheat species and varieties were grown in the same trial field in plots of 4.5 m^2^. At maturity, approximately 50 ears of each sample were harvested from each plot. The wheat samples collected were all spring types, including the ancestral wheat species einkorn (diploid, AA), emmer (tetraploid, AABB) and spelt (hexaploid, AABBDD), as well as four selected varieties of common wheat (hexaploid, AABBDD). These seven different wheat varieties (Table 1) are hereafter referred to as “wheat types”. The harvested wheat was dried to below 15% moisture at 30 °C for 3 days before threshing and cleaning (Perten Instruments AB, Hägersten, Sweden).

### 2.2. Wheat Characterization

Einkorn, emmer and spelt were hulled manually after threshing. The samples were milled to whole meal flour by Falling Number Laboratory 3100 with a 0.8 mm screen (Perten Instruments AB, Hägersten, Sweden) before further analysis. Kernel size was recorded as weight per thousand kernels (TKW). Grains were counted by an Elmor C1 seed counter (Elmor Ltd., Schwyz, Switzerland), and presented as weight in grams per thousand grains. The moisture content of the grain was determined by drying kernels for 24 h at 105 °C. Further, the nitrogen content of the wheat samples was measured by the micro Kjeldahl method (Kjeltec™ 8400, Tecator, Foss, Hillerød, Denmark), and wheat protein content was determined using 5.7 as the Kjeldahl factor. Total starch content was analyzed by using the Megazyme kit (K-TSTA-100A 08/19, Megazyme, Bray, Ireland) [20]. The porridge was prepared by mixing whole wheat flour and water (1:20 w/v), which was then heated at 100 °C in a water bath for 10–15 min, homogenized, cooled and stored at 4 °C until ex vivo digested.

### 2.3. Ex vivo Digestion of Wheat Porridge

Human gastric and duodenal juices were collected according to Ulleberg et al. [21] by aspiration of self-reported healthy volunteers (*n* = 20) at Lovisenberg Diaconal Hospital, Norway. All subjects reported no CeD symptoms and gave their informed consent for inclusion before participation. The aspiration was approved by the Regional Committees for Medical and Health Research Ethics (REK 2012/2230 and 2012/2210) in Norway. In short, a flexible three-lumen silicone tube was placed through the nose or mouth into the gastric antrum and duodenum, using gastroscopic guidance. An isotonic stimulatory solution (17.5 g/L sucrose, 450 mg/L NaCl, 800 mg/L L-phenylalanine and 575 mg/L L-valine in H_2_O) was continuously infused (100 mL/h) simultaneously as the gastric and duodenal fluids were aspirated. The aspirates were pooled and stored at −20 °C, then at −80 °C [21].

The enzymatic activity of pepsin and trypsin was assayed according to Minekus et al. [22]. Digestion with human GI enzymes was performed according to the standardized INFOGEST consensus model [22] with some modifications. A porridge aliquot (1 g with approximately 5 mg/mL protein) was mixed 1:1 (w/v) with salivary fluid (SSF) containing α-amylase (75 U/mL, Sigma Aldrich) and incubated for 2 min, simulating the oral phase. The gastric digestion phase was performed by adding simulated gastric fluid (SGF) with human gastric juices (HGJ) (2000 U/mL pepsin activity) to the oral phase (1:1, v/v) and adjusting the pH to 3.0 by the addition of 1 M HCl. The samples were incubated in a water bath at 37 °C with gentle magnetic stirring for 120 min. The duodenal digestion phase was done by adding simulated intestinal fluid (SIF) containing human duodenal juice (HDJ) (100 U/mL trypsin activity) to the gastric sample (1:1 v/v). The pH was adjusted to 7.0 by the addition of 1 M NaOH and the samples were incubated in a water bath at 37 °C for another 120 min with magnetic stirring, then terminated by adding 5 mM Pefabloc^®^ (Sigma Aldrich, St. Louis, MO, US). The digestion was performed in parallel and all samples were immediately stored at −20 °C until further analysis.

### 2.4. Peptide Profile by HPLC-ESI MS/MS 

Prior to HPLC-ESI MS/MS analysis, digests (100 µL) were desalted using a C18 spin column (Thermo Scientific, San Jose, CA, USA), according to the manufacturer’s instructions, eluting with 70% acetonitrile (v/v)/0.1% trifluoroacetic acid (TFA). MS analysis was performed using a Q Exactive Orbitrap mass spectrometer (Thermo Scientific, San Jose, CA, USA), online coupled with an Ultimate 3000 ultra-high-performance liquid chromatography instrument (Thermo Scientific, San Jose, CA, USA). Purified peptides were diluted in 50 µL of 0.1% (v/v) formic acid solution, loaded through a 5 mm long, 300 mm internal diameter pre-column (LC Packings, San Jose, CA, USA) and separated by an EASY-Spray™ PepMap C18 column (2 µm, 15 cm–75 µm; 3 mm particles; 100 Å pore size (Thermo Scientific, San Jose, CA, USA)). Eluent A was 0.1% formic acid (v/v) in Milli-Q water and eluent B was 0.1% formic acid (v/v) in acetonitrile. The column was equilibrated with 5% eluent B. Peptides were separated by a 4–40% eluent B gradient over 60 min (300 nL/min). The mass spectrometer operated in data-dependent mode and all MS1 spectra were acquired in the positive ionization mode by scanning the 1800–350 *m/z* range. A maximum of 10 of the most intense MS1 ions were fragmented in MS/MS mode. The resolving power was set at 70,000 full width at half maximum (FWHM), using automatic gain control (AGC) target of 1 × 10^6^ ions and 100 ms as a maximum ion injection time (IT) to generate precursor spectra. MS/MS fragmentation spectra were obtained at a resolving power of 17,500 FWHM and 10 s dynamic exclusion was used to prevent repeated fragmentation of the most abundant ions. Ions with one or more than six charges were excluded from fragmentation. Spectra were elaborated using the Xcalibur Software 3.1 version (Thermo Scientific, San Jose, CA, USA).

### 2.5. MS Analysis Spectra Identification

Peptides were identified from the MS/MS spectra using the Proteome Discoverer 2.1 software (Thermo Scientific, San Jose, CA, USA), based on the Sequest searching algorithm. Searches were taxonomically restricted to the *Triticum* database extracted from UniProtKB (downloaded in February 2018). Search parameters were: Met oxidation and pyroglutamic acid for N-terminus Gln as variable protein modifications; a mass tolerance value of 10 ppm for precursor ions and 0.01 Da for MS/MS fragments; no proteolytic enzyme selected. The false discovery rate and protein probabilities were calculated by a target decoy peptide spectrum match (PSM) validator working between 0.01 and 0.05 for strict and relaxed searches, respectively. Data from three replicate LC-MS/MS analyses were merged. Peptide amount was inferred by the number of PSMs and the relevant ion count.

The T cell epitopes were determined by their native gliadin sequences as they appear prior to deamidation because tTG2 treatment was not included in the current study. These peptides are expected to be modified in vivo by lamina propria tTG2 and become immunogenic as tTG2 deamidate glutamine (Q) to glutamate (E). Only deamidated peptides fit in the HLA-DQ2.5/8 peptide binding groove and stimulate CeD promoting CD4+ T cells [23,24].

## 3. Results

Protein content varied from approximately 8.2% to 11%, and all ancestral wheat types showed values above 10% protein (Table 2). The starch content was in the standard range (55% to 66%) for all wheat types and the thousand-kernel weight (TKW) varied from 30 to 41 g between the different wheat types.

**Figure 1:**
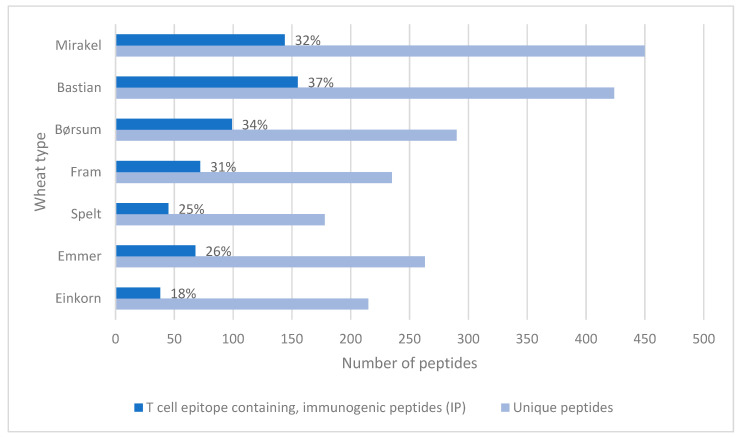
Representation of unique peptides identified in each wheat type and the number of T cell reactive epitopes in the unique peptides, according to Sollid, Qiao [19]. Putative immunogenic peptides are presented as a percentage of total unique peptides within each wheat type (identified by HPLC-ESI MS/MS).

**Figure 2:**
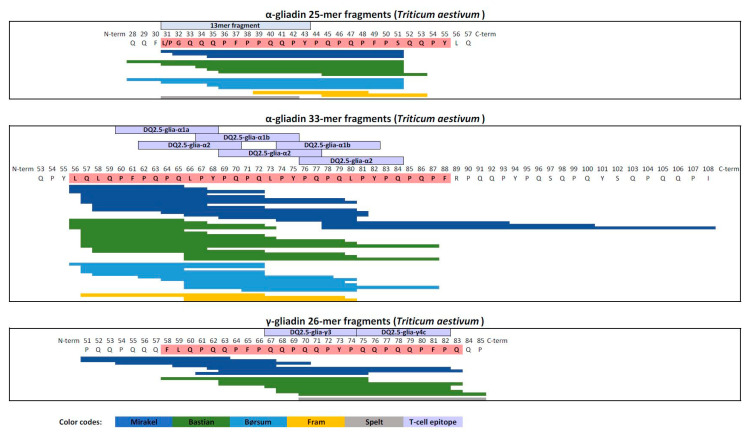
Peptides identified in the hexaploid wheat digests which correspond to alpha-gliadin 33-mer and 25-mer, and gamma-gliadin 26 mer. Different wheat types are represented by different colours: Mirakel, dark blue; Bastian, green; Børsum, light blue; Fram, yellow; spelt, grey. Known T cell epitopes within the illustrated sequences are presented in purple above the sequence.

Ex vivo digestion of porridge samples with human GI juices produced a complex variety of gluten protein fragments, which were identified by HPLC-ESI MS/MS and software-based matching. Overall, 1051–2689 peptides were identified for each sample. An assorted list of non-redundant unique peptide sequences was generated and used to identify T cell reactive epitopes in the reference list [19]. Whereas spelt digestion released few peptides, it gradually increased in einkorn (diploid) and emmer (tetraploid), and further in the common hexaploid wheat varieties Fram, Mirakel and Bastian. Thus, the latter two varieties had the highest number of unique peptides, of which 144 and 155 were T cell epitope-containing immunogenic peptides (IPs), respectively (Figure 1). This is in contrast to einkorn, spelt and emmer, which released only 38, 45 and 68 IPs, respectively (Figure 1). Thus, digesting einkorn released considerably less IPs (18% of the peptides) than Bastian (37% of the peptides). Interestingly, none of the digested wheat types contained intact gliadin-33mer, -26mer or -25mer (Figure 2).

**Figure 3:**
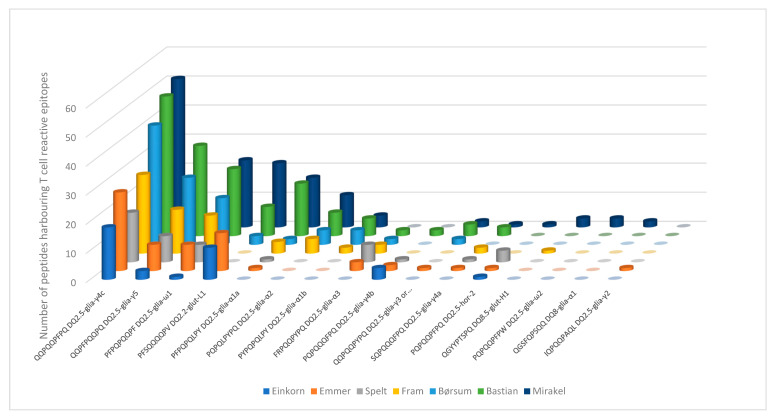
Graphical representation of the number of peptides with the indicated T cell epitope in each wheat type represented by different colors. The number of epitopes is retrieved by counting the given epitope sequences in the assorted peptide list. Each bar represents a naïve T cell epitope sequence, e.g., the PFPQPQLPY sequence is specific to the DQ2.5-glia-α1a epitope [19]. Abbreviations are used to denote which gliadin fraction the epitopes are derived from: glia-α, α-gliadin; glia-γ, γ-gliadin; glia-ω, ω-gliadin; glut-L, low molecular weight glutenin; glut-H, high molecular weight glutenin; hor, hordein.

Although few peptides perfectly matched the T cell reactive epitopes in the reference list [19], the larger peptides contained these IP sequences. Digests of all wheat types contained peptides with the DQ2.5-glia-γ4c (QQPEQPFPQ), DQ2.5-glia-γ5 (QQPFPQQPQ) and DQ2.5-glia-ω1 (PFPQPQQPF) epitopes, whereas only the hexaploid (*T. aestivum*) wheat types, (Fram, Børsum, Bastian and Mirakel) released peptides with the DQ2.5-glia-α2 (PQPELPYPQ) and DQ2.5-glia-α1b (PYPQPQLPY) epitopes (Figure 3). In particular, einkorn released fewer IPs with less variation, as only six of the 22 T cell reactive epitopes were detected. This in contrast to spelt, Bastian and Mirakel, which released eight, 11 and 13 different T cell reactive epitopes, respectively. However, the DQ2.5-hor-2 (PQPQQPFPQ) and DQ2.5-glia-γ4b (PQPQQQFPQ) epitopes dominated in ancestral wheat digests, as they were only present in one and two peptides from the common wheat types, respectively.

**Figure 4:**
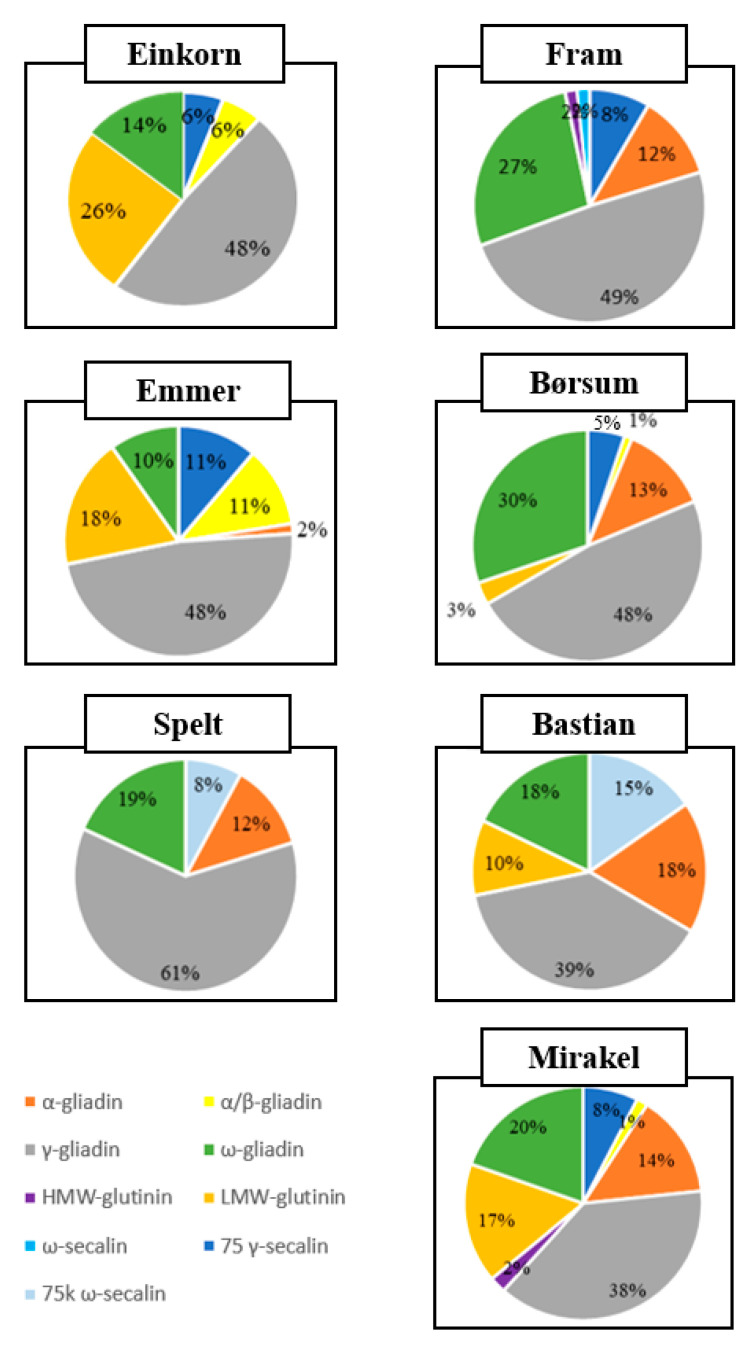
Diagrammatic representation of the percentage of T cell epitopes from individual parent protein subfamilies. Each protein fraction combined several different peptides with various access numbers from UniprotKB. LMW, low molecular weight; HMW, high molecular weight.

γ-Gliadins released the highest number of T cell epitope-containing peptides in all wheat types, as illustrated in Figure 4. Low molecular weight glutenins were the second largest contributor of IPs in einkorn and emmer; ω-gliadins ranked as the second largest contributor, followed by α-gliadins in the common hexaploid wheat types. Only Fram and Mirakel released some IPs from high molecular weight glutenins. In addition, we also observed T cell epitope-containing peptides from wheat secalins in all wheat types, which are proteins commonly found in rye and most likely identified here by homology. 

## 4. Discussion

This study aimed to characterize CeD-associated T cell epitopes in peptides released during ex vivo gastrointestinal digestion of three ancestral wheat types (einkorn, emmer and spelt), and four common Norwegian wheat varieties (Fram, Børsum, Bastian and Mirakel). Similar to Shan et al. [10], several studies have established that the digestive-resistant 33mer (LQLQPFPQPQLPYPQPQLPYPQPQLPYPQPQPF), and 25mer (LGQQQPFPPQQPYPQPQPFPSQQPY) fragments from α-gliadin [25], and the 26mer (FLQPQQPFPQQPQQPYPQQPQQPFPQ) from γ-gliadin [26] contain most of the T cell reactive epitopes involved in the CeD immune reaction [27]. Surprisingly, these large immunodominant peptides were cleaved into smaller, but still T cell epitope-containing peptides by the current ex vivo digestive systems. This is in contrast to previous in vitro digestion experiments showing that the 33mer, 26mer and 25mer peptides were digestion resistant. Whereas previous digestive models used bovine or porcine digestive enzymes, the current experiments were performed with human gastroduodenal aspirates that increase the physiological relevancy of the model, and therefore more closely mimic in vivo gastrointestinal digestion [28]. However, as we used gastroduodenal aspirates from healthy, apparently non-celiac controls, we cannot rule out the possibility that celiac patients digest gluten differently due to either genetic variations in intestinal digestive enzymes or to differences in microbial-assisted gluten digestion.

The digested wheat peptides contained both single and multiple overlapping immunogenic core sequences. Whereas einkorn released the lowest number of possible Ips, followed by spelt, Bastian released the highest number of IPs. Although the percentage of IPs to the total number of peptides did not vary significantly between wheat types with different genomes, the ancestral varieties generally had a lower percentage of IPs. This is in contrast to Prandi et al. [29], who reported that the in vitro digestion of old wheat varieties produced more IPs compared to modern varieties. However, their modern wheat samples included both einkorn and spelt, which in our study were classified as ancestral, and their old varieties included only tetraploid and hexaploid wheat varieties (*T. aestivum* L., *T. turgidum* var. *durum* Desf., *T. turgidum* var. dicoccum L (emmer)), which makes it difficult to compare results. Our findings are also in contrast to Malalgoda et al. [30], who observed no differences between historical and modern wheat cultivars in T cell epitope-containing peptides released after in-gel gliadin digestion with porcine chymotrypsin. However, this in-gel digestion system with commercial enzymes does not mimic human gastrointestinal digestion as well as the current ex vivo digestive system does using human gastroduodenal juices.

The α-gliadin 33mer gene loci is located on chromosome 6D in the hexaploid wheat (AABBDD) varieties only [31]. Thus, the 33mer sequence is lacking in einkorn (AA) and emmer (AABB) but may be present in spelt, Fram, Børsum, Bastian and Mirakel (AABBDD). The α-gliadin 33mer pepride harbors six overlapping T cell epitope sequences: one copy of the DQ2.5-binding gliadin peptide, glia-α1a (PFPQPQLPY), two copies of the DQ2.5-glia-α1b (PYPQPQLPY) and three copies of the DQ2.5-glia-α2 (PQPQLPYPQ). Whereas none of these epitopes were detected in the diploid einkorn, digested emmer and spelt released one peptide with the DQ2.5-glia-α1a epitope. In contrast, these T cell epitopes were present in many peptides from the hexaploid wheat varieties. In particular, Mirakel, Bastian and Børsum released several 33mer fragments (Figure 2). The chromosome 6D-derived gliadins were cleaved at different positions, producing peptides of different lengths with similar or multiple overlapping epitope sequences. Thus, digestion of these wheat varieties released more T cell epitope-containing peptides than the diploid and tetraploid wheat types that lack chromosome 6D.

The γ-gliadin-derived 26mer peptide contains two overlapping T cell epitopes, the DQ2.5-glia-γ3, (QQPQQPYPQ) and the DQ2.5-glia-γ4c (QQPQQPFPQ). Whereas the DQ2.5-glia-γ3 epitope was absent in einkorn and spelt, it was present in digests from the other hexaploid wheat types and emmer. Although the DQ2.5-glia-γ4c epitope was the most dominant peptide epitope in all the digested wheat samples, it occurred in variable quantities, from a moderate amount in einkorn and spelt, to almost a three-fold increase in the common wheat varieties. The other dominant epitopes, DQ2.5-glia-γ5 (QQPFPQQPQ) and DQ2.5-glia-ω1 (PFPQPQQPF), together with DQ2.5-glia-γ4c, were also present after complex proteolytic digests of gluten using human monoclonal antibody pull-down techniques to identify the epitopes [32].

The α-gliadin 25mer peptide was undetected in all samples. However, einkorn, emmer, Børsum, Bastian and Mirakel, but not spelt or Fram, released a shortened 20mer (_31_LGQQQPFPPQQPYPQPQPFPS_51_) peptide. Several studies have shown that the 13mer _31_LGQQQPFPPQQPY_43_ sequence within the α-gliadin 25mer peptide and the shortened 20mer peptide detected in this study, activates the innate immune system [33] by upregulating interleukin-15, cyclooxygenase-2 (COX-2), CD25 and CD83 expression on lamina propria macrophages, monocytes, and dendritic cells prior to any CD4+ T cell stimulation [34]. More recently, Barone et al. [35] showed that the 13mer induced altered vesicular trafficking in the colonic epithelial cancer cell line Caco-2. This lead to overexpression of trans-presented IL-15/IL5R alpha complex that induced an EGFR-dependent cell proliferation that may explain the mucosal remodeling in CeD. Thus, innate immune system may also be involved in this aspect of the CeD pathogenesis.

## 5. Conclusions

The ex vivo human gastrointestinal digestion of diploid, tetraploid and hexaploid wheat types produced T cell epitope-containing peptides depending on the genomic asset. Wheat digestion with human gastrointestinal juices produced a different protein degradation pattern compared to previously reported studies that used enzymes of porcine or bovine origin. In our study, the immunodominant peptides (the 33mer and 25mer α-gliadin peptides, and the 26mer γ-gliadin peptide) were not found in their intact form, but as degraded fragments. The present digestion model did not include mucosal degradation and absorption. Therefore, the bioaccessibility and the capability of these peptides to reach the lamina propria and bind tTG2 through deamidation, and thereby acquiring HLA-DQ2.5/8 binding properties and becoming T cell epitopes, remains to be studied. The results suggested, nevertheless, that ancestral wheat types may be less CeD toxic compared to the common hexaploid varieties. Whether more general use of these ancestral wheat variants in genetically predisposed individuals could reduce CeD needs further assessment, but the incidence of diagnostic CeD in childhood has been linked to the amount of gluten in the diet [36].

## Figures and Tables

**Table 1:** Genomic asset and origin of the wheat types studied.

Wheat Type	Species	Genome	Variety	Breeding Company/Origin	Marked Release
Einkorn	*T. monococcum*	AA	Unknown		
Emmer	*T. dicoccon*	AABB	Gotland		
Spelt	*T. aestivum var. spelta*	AABBDD	Vit Gotland		
Common wheat	*T. aestivum var. aestivum*	AABBDD	Fram	Norwegian landrace	Before 1900
		AABBDD	Børsum	Norwegian Agricultural University (NLH)	1936
		AABBDD	Bastian	Graminor, Norway	1989
		AABBDD	Mirakel	Graminor, Norway	2012

**Table 2:** Variations in grams per thousand kernel (TKW), starch and proteins among the different wheat types (einkorn, emmer and spelt) and the common wheat types (Fram, Børsum, Bastian and Mirakel).

Wheat Sample	TKW (g)	Protein (%)	Starch (%)
Einkorn	30.5	10.3	66.5
Emmer	31.7	11.0	50.0
Spelt	41.1	10.6	60.0
Fram	32.3	8.2	63.1
Børsum	31.9	9.2	55.6
Bastian	33.1	10.2	58.8
Mirakel	38.5	9.1	63.4
